# Melanoma Incidence and Mortality Trends Among Patients Aged 59 Years or Younger in Sweden

**DOI:** 10.1001/jamadermatol.2024.3514

**Published:** 2024-09-08

**Authors:** Hildur Helgadottir, Rasmus Mikiver, Karina Schultz, Kari Nielsen, Francesca Portelli, Jan Lapins, Susana Puig, Karolin Isaksson

**Affiliations:** 1 Department of Oncology and Pathology, Karolinska Institutet, Stockholm, Sweden; 2 Theme Cancer, Karolinska University Hospital, Stockholm, Sweden; 3 Regional Cancer Center Southeast Sweden and Department of Clinical and Experimental Medicine, Linköping University, Linköping, Sweden; 4 Department of Dermatology, Skåne University Hospital, Lund, Sweden; 5 Department of Clinical Sciences, Dermatology, Lund University Skin Cancer research group, Lund University, Lund, Sweden; 6 Department of Dermatology, Helsingborg Hospital, Helsingborg, Sweden; 7 Department of Clinical Pathology and Cancer Diagnostics, Karolinska University Hospital, Stockholm, Sweden; 8 Department of Dermatology, Karolinska University Hospital, Stockholm, Sweden; 9 Dermatology and Venereology Unit, Department of Medicine Huddinge, Karolinska Institutet, Stockholm, Sweden; 10 Melanoma Unit, Dermatology Department, Hospital Clinic de Barcelona, Institut d’Investigacions Biomèdiques August Pi i Sunyer, Universitat de Barcelona, Barcelona, Spain; 11 Centro de Investigación Biomédica en Red de Enfermedades Raras, Instituto de Salud Carlos III, Barcelona, Spain; 12 Department of Clinical Sciences, Surgery, Lund University, Lund, Sweden; 13 Department of Surgery, Kristianstad Hospital, Kristianstad, Sweden

## Abstract

**Importance:**

Over the past decades, many global regions have experienced a steady increase in the incidence of cutaneous melanoma. However, more recently, a downward trend has been observed in the younger age groups in Australia and the US. Yet, in Europe, none of the countries have reported any significant decline in melanoma incidence for any age group.

**Objective:**

To assess melanoma incidence and mortality trends in Sweden, with a focus on individuals younger than the average age of melanoma onset.

**Design, Setting, and Participants:**

This cohort study used data on the national population from the Swedish Melanoma Registry and the Swedish Cancer Registry, which cover more than 99% of all primary invasive cutaneous melanomas diagnosed in the country. All patients diagnosed from 1990 to 2022 were included.

**Main Outcomes and Measures:**

Incidence and mortality rates per 100 000 inhabitants were calculated for each year and shown as average annual rates for every 5-year period from 1990 to 2022. Joinpoint regression models were used to evaluate statistical significance of temporal trends and points of change.

**Results:**

There were 34 800 primary invasive cutaneous melanomas (19 582 [56.3%] in females and 15 218 [43.7%] in males) reported in 33 324 individuals younger than 60 years (median [IQR] age, 48 [36-58] years) from 1990 to 2022. A consistent rise in melanoma incidence was observed among those 50 to 59 years old. The age groups from 20 to 29 years, 30 to 39 years, and 40 to 49 years showed an incidence peak in 2013 to 2015 followed by stable or significantly declining rates until 2022. In patients younger than 20 years, melanoma incidence remained low with no significant trends. There was also a significant decline in melanoma mortality among 30- to 59-year-old individuals, but not in those 60 years and older.

**Conclusions and Relevance:**

The findings of this cohort study showed a significant recent downward trend in both melanoma incidence and melanoma mortality in the age group 30 to 49 years in Sweden. The reasons for these declines are unclear but may include UV protection, public health campaigns, changing population demographics, and the introduction of effective melanoma treatment. None of these possibilities were evaluated; further study is needed.

## Key Points

**Question:**

How have melanoma incidence and mortality rates evolved in the population younger than 60 years in Sweden?

**Findings:**

This cohort study including the entire population of Sweden found a continuous rise in primary invasive cutaneous melanoma incidence among individuals aged 50 to 59 years from 1990 to 2022; however, those aged 20 to 49 years experienced peak incidence in 2013 to 2015 followed by stable or statistically significant declining rates through 2022, while in patients younger than 20 years, incidence remained low (unchanged). A statistically significant decline in melanoma mortality was seen in 30- to 59-year-olds, but not in those 60 years or older.

**Meaning:**

These findings suggest that melanoma incidence and mortality may eventually decrease among the entire population in Sweden.

## Teaser

This cohort study assesses incidence of primary invasive cutaneous malignant melanoma and mortality trends in Sweden, focusing on the population younger than the mean age of melanoma onset.

## Introduction

Since the 1960s, there has been a well-documented and steady increase in the incidence of primary invasive cutaneous malignant melanoma (hereafter, melanoma) throughout Europe, North America, and Oceania.^1,2,3,4,5^ The increased incidence has been most prominent among older individuals, and although it has remained low in prepubertal children, there have been reports of rising rates among them as well as among adolescents and young adults.^1,6,7,8^ However, in recent years, Australia and the US have experienced a downward trend in melanoma incidence in younger age groups.^1,8,9^ Furthermore, in some countries—eg, Canada, New Zealand, Norway, Spain, and the United Kingdom—an incidence stabilization has been reported in younger age groups.^1,2,5,6,7,9,10,11,12^ Yet, none of the European countries have reported a significant decline in melanoma incidence in any age group. The mortality from melanoma on a population level has increased in many countries, but at a substantially slower rate than melanoma incidence.^5,13,14^ Furthermore, in several countries, a decrease in melanoma mortality among younger individuals has been reported.^5,9,15^

In this study, we used the highly comprehensive Swedish national health and population registers to explore melanoma trends among those diagnosed at younger than the average age of melanoma diagnosis, including children, adolescents, and young and middle-aged adults. The median age of diagnosis of invasive melanoma in Sweden has been on the rise—in 1990, it was 60 years and it is currently 65 years.^4,11,16^ In the Swedish population as a whole, there has been no leveling off in melanoma incidence rise that during the past decade has been approximately 6% annually, with a proportionately steeper increase among older individuals who also tend to be diagnosed with thicker tumors.^14,16^ Melanoma incidence in Sweden is the sixth highest globally, preceded by Australia, New Zealand, Denmark, the Netherlands, and Norway.^17^ Sweden currently has a population of 10.5 million inhabitants and an annual age standardized (world) melanoma incidence of 23.3 per 100 000 inhabitants. In this study, we assess national-level data on melanoma incidence and mortality from 1990 to 2022 among those younger than 60 years.

## Methods

This study was reviewed and approved by the Swedish Ethical Review Authority (2023-02006-01). Information on patients and tumors was derived from national registries. According to Swedish law, all diagnosed malignant tumors are registered with the National Board of Health and Welfare and the Regional Cancer Centers; patient consent is not collected.

### Data Collection

This cohort study on primary invasive cutaneous melanoma in younger individuals is based on the national population based Swedish Melanoma Registry (SweMR) and on the national Swedish Cancer Registry (SCR). In situ tumors, noncutaneous melanomas, or melanoma metastasis with unknown primary were not included. By Swedish law, clinicians and pathologists are obliged to report all cancer diagnoses to SCR; however, this registry contains limited information on individual diagnoses.^18^ On the other hand, SweMR collects several clinicopathologic parameters and therefore, provides detailed information on all individual reported cases of invasive melanomas. The SCR was started in 1958, and SweMR inception was in 1990; however, due to some of the nation’s regional differences in starting the registration, SweMR coverage was not complete until 1996. From 1990 to 1995, 86% of all melanomas reported to SCR were also registered to SweMR. Subsequently, since 1996, SweMR has included all of Sweden’s regions, covering nearly all (99%) primary invasive cutaneous melanomas diagnosed and reported to SCR and providing nearly complete coverage on a national level.^19^ Information on deaths from melanoma was collected from the national Cause of Death Registry, a high-quality nearly complete register of all deaths in Sweden.^20^ For this study, mortality data from 1997 were used because that was the year when the Cause of Death Registry started using the *International Statistical Classification of Diseases and Related Health Problems, Tenth Revision*, with its more detailed information on death causes; it is still in use.

### Study Population and Variables

This study included patients who were younger than 60 years on the date of surgical treatment for a histologically confirmed primary invasive cutaneous melanoma in 1990 to 2022. Separate analyses were performed for sex, age group, and Breslow thickness (≤1 mm or >1 mm) of the melanoma. Age groups were defined as 20 to 29, 30 to 39, 40 to 49, and 50 to 59 years. Although the age group of those 60 years and older was not included in the study, this group was included for reference in the mortality analyses.

### Statistical Analyses

Incidence and mortality rates per 100 000 inhabitants were calculated for each year and shown as average annual rates for every 5-year period. The Swedish population denominators were derived from the yearly census for the Swedish population, including all residents in the country. Data from the SCR (from 1990 onwards) was used for the analyses of incidence trends, independent of melanoma thickness. For the incidence of melanomas with thickness of 1 mm or less or greater than 1 mm, we used data from the SweMR (from 1996 onwards). The age groups defined to study the trends in melanoma incidence were diagnosis at age 0 to 19, 20 to 29, 30 to 39, 40 to 49, or 50 to 59 years. Mortality trends were assessed from 1997 and analyzed for the age groups 0 to 29, 30 to 49, and 50 to 59 years.

Joinpoint regression models were used to evaluate whether temporal trends had statistically significant points of change, and to estimate the average annual percentage change (AAPC), with subanalyses by sex, age groups, and tumor thickness. The time periods were autogenerated by the joinpoint analyses, and for each time period, the AAPC was calculated using Joinpoint Regression Software (Surveillance Research Program, the US National Cancer Institute).^21,22^ This Joinpoint regression program used a permutation test to find the optimal number of joinpoints. The analyses allowed a maximum of 2 joinpoints, and the maximum number of estimated time periods was consequently 3.

The statistical analyses were performed using IBM SPSS Statistics, version 29 (IBM Corp); R Statistical Software, version 4.0.3 (The R Foundation for Statistical Computing); and Joinpoint Trend Analysis Software, version 5.0.2 (US National Cancer Institute). Statistical tests were 2-tailed and *P* values < .05 were considered statistically significant.

## Results

There were 34 800 primary invasive cutaneous melanomas (19 582 [56.3%] in females and 15 218 [43.7%] in males) registered in SweMR and/or SCR in 33 324 individuals younger than 60 years (median [IQR] age, 48 [36-58] years) from 1990 to 2022 (Table). Of the 91 123 total primary invasive cutaneous melanomas diagnosed in the whole Swedish population,^14^ those diagnosed in patients younger than 60 years comprised 38.1%, and among those younger than 50 years, 21.5%. In 1476 cases (4.4%), more than 1 invasive primary melanoma was diagnosed before age 60. Few melanomas were diagnosed in the youngest age groups and a steep increase was observed for each additional decade in age.

**Table.** Primary Invasive Cutaneous Melanomas Reported (n = 34 800) in 33 324 Patients Younger Than 60 Years, Sweden, 1990 to 2022, by Age

Characteristic	Age group, No. (%)					
	0-12 y	13-19 y	20-29 y	30-39 y	40-49 y	50-59 y
Male patients, No.	9	91	794	2289	4504	7531
Breslow thickness^a^						
≤1 mm	1 (16.7)	40 (46.5)	477 (65.3%)	1402 (66.0)	2707 (64.6)	4252 (60.4)
>1 mm	5 (83.3)	46 (53.5)	253 (34.7)	722 (34.0)	1483 (35.4)	2789 (39.6)
Missing data (in SweMR)	0	0	22	64	103	207
Missing in SweMR (only in SCR)^b^	3	5	42	101	211	283
Female patients, No.	9	166	1604	3696	6454	7653
Breslow thickness^a^						
≤1 mm	1 (20.0)	101 (68.7)	1108 (74.1)	2496 (70.9)	4323 (71.6)	4802 (66.5)
>1 mm	4 (80.0)	46 (31.3)	388 (25.9)	932 (27.2)	1715 (28.4)	2417 (33.5)
Missing data (in SweMR)	2	10	44	93	167	193
Missing in SweMR (only in SCR)^b^	2	9	64	175	249	241

Abbreviations: SCR, Swedish Cancer Registry; SweMR, Swedish Melanoma Registry.

^a^ Breslow thickness is not available in cases only identified through SCR.

^b^ Patients only in the SCR and not in the SweMR, the majority (81%) in 1990-1995, because registration in the SweMR had not started in all regions.

Only 18 melanomas were diagnosed in children aged 0 to 12 years, and 257 in those aged 13 to 19 years. While there were similar numbers of melanomas in each of the sexes in the youngest (age 0-12 years) and oldest (age 50-59 years) subgroups, there was a clear overrepresentation of females among those diagnosed at age 13 to 49 years. This was most evident in the 20- to 29-year-old group, in which the number of females (n = 1604) was more than twice that of males (n = 794). From age 20 to 49 years, both females and males had melanomas that tended to be thinner (≤1 mm) compared with those aged 0 to 19 years and 50 to 59 years. Given that the numbers of melanomas in the children (age 0-12 years) were so low (mean, 1 melanoma every 2.5 years), they were combined with 13- to 19-year-olds for the incidence analyses.

### Incidence Trends by Age Group

**Figure 1.**
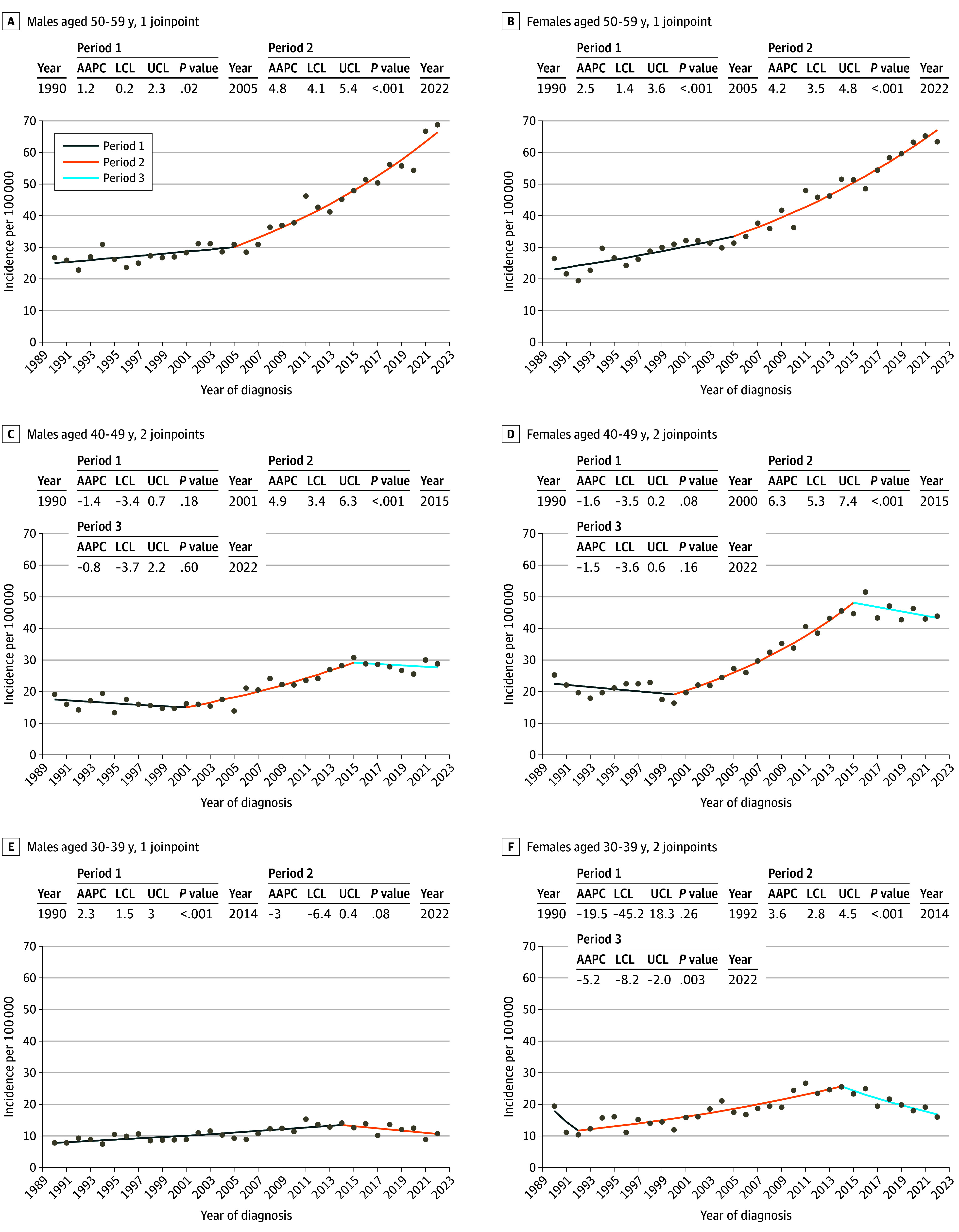
Average Annual Percentage Change (AAPC) in the Primary Invasive Cutaneous Melanoma Incidence in Sweden From 1990 to 2022, by Age Group Based on melanomas registered to the Swedish Cancer Registry in 1990 to 2022. The year indicates the year of start and end of each period; LCL, lower confidence limit; and UCL, upper confidence limit.

In the age group 50 to 59 years, for females and males, there was a consistent melanoma incidence increase observed throughout the period, with an incline in the steepness of the curve and a significant joinpoint in 2005 (Figure 1; and eFigure 1 in Supplement 1). The AAPC for this age group post-2005 was more than 4%, with no tendency to level off and melanoma incidence is currently 65 cases per 100 000, similar in both sexes (AAPC in the females, 4.2%; 95% CI, 3.5-4.8;* P *< .001; and in males, 4.8%; 95% CI, 4.1-5.4; *P* < .001).

In 40- to 49-year-olds, there was no incidence increase until 2000, when the AAPC rose to 6.3% (95% CI, 5.3 to 7.4;* P *< .001) and 4.9% (95% CI, 3.4 to 6.3;* P *< .001) in females and males, respectively (Figure 1). Interestingly, after 2015, there was a leveling off for both females (AAPC −1.5%; 95% CI, −3.6 to 0.6;* P *= .16) and males (AAPC −0.8%; 95% CI, −3.7 to 2.2;* P *= .59) in this age group. At the end of the study period, annual melanoma incidence in 40 to 49-year-olds was 45 and 25 per 100 000 in females and males, respectively.

In the age group 30 to 39 years, there was an incidence peak in 2014; thereafter, a significant decrease was observed in females, with an AAPC of −5.2% (95% CI, −8.2 to −2.0;* P *= .003) (Figure 1). In the 30- to 39-year-old males, a similar trend was seen with an AAPC of −3.0% (95% CI, −6.4 to 0.4;* P *= .08). By the end of the study period, melanoma incidence in those aged 30 to 39 years was 18 and 10 per 100 000 in females and males, respectively.

Also, among 20- to 29-year-old females a peak was seen in 2014 and thereafter, a significant decrease with an AAPC −5.2% (95% CI, −9.0 to −1.3;* P *= .01) (eFigure 2 in Supplement 1). In males aged 20 to 29 years there were no significant changes or joinpoints in the incidence in the years 1990 to 2022. By the end of the study period, melanoma incidence in 20- to 29-year-olds was 7 and 3 per 100 000, in females and males, respectively.

In the youngest age group (combined, 0 to 19 years) there were no significant joinpoints in incidence from 1990 to 2022, and the incidence remained low—well below 1 per 100 000 for both sexes (eFigures 2 and 3 in Supplement 1). In males aged 0 to 19 years, there was no significant incidence change during the study period, whereas, among females in this age group, there was a significant decrease in incidence, with an AAPC of −2.1% (95% CI, −3.7 to −0.4;* P *= .019).

### Incidence Trends by Tumor Thickness

**Figure 2.**
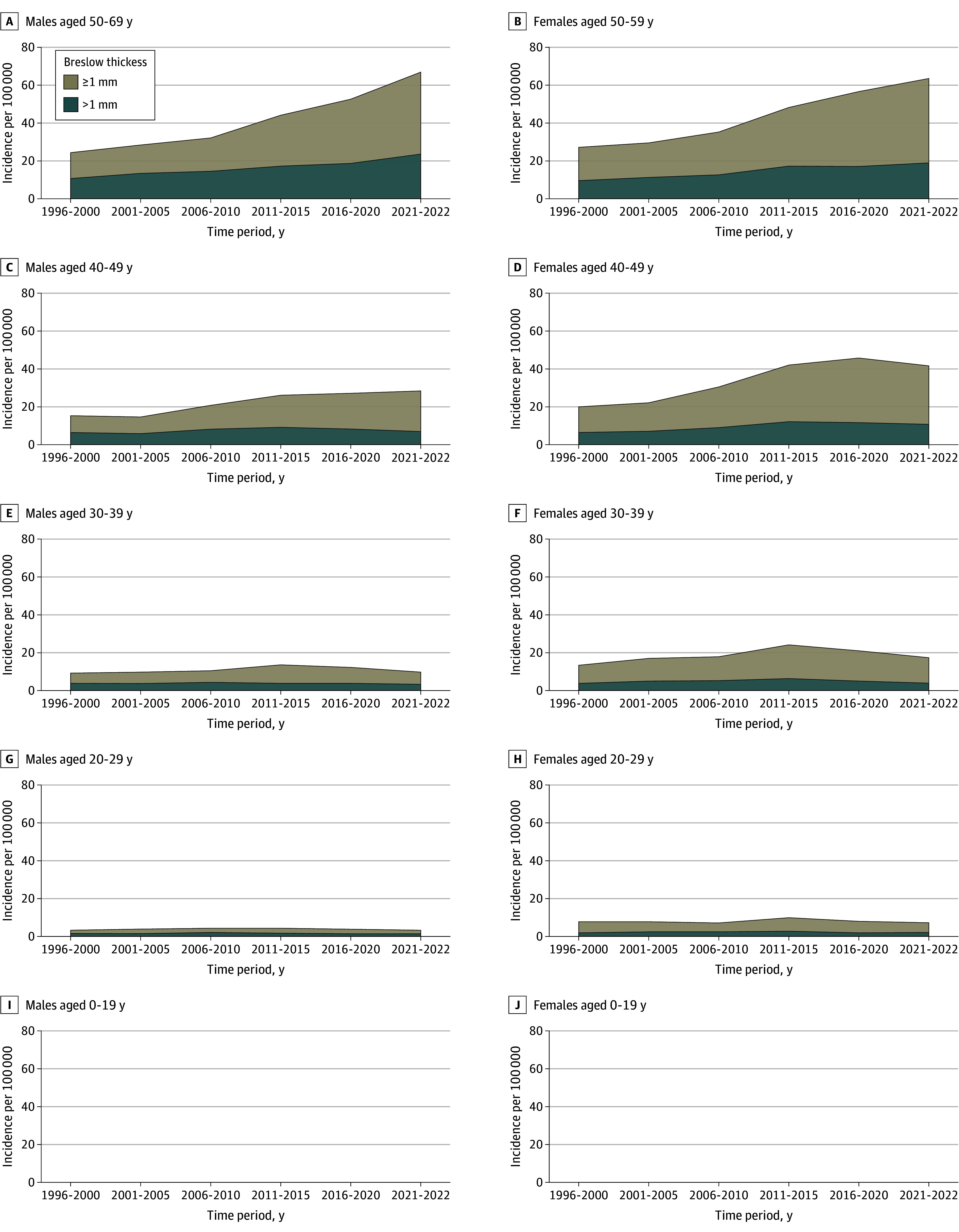
Incidence Trends in Cutaneous Melanomas (Breslow Thickness ≤1 mm or >1 mm) in Sweden From 1996 to 2022 Based on melanomas registered to the Swedish Melanoma Registry in 1996 to 2022 and shown as the average incidence per 5-year period, except for the 2-year period from 2021 to 2022.

**Figure 3.**
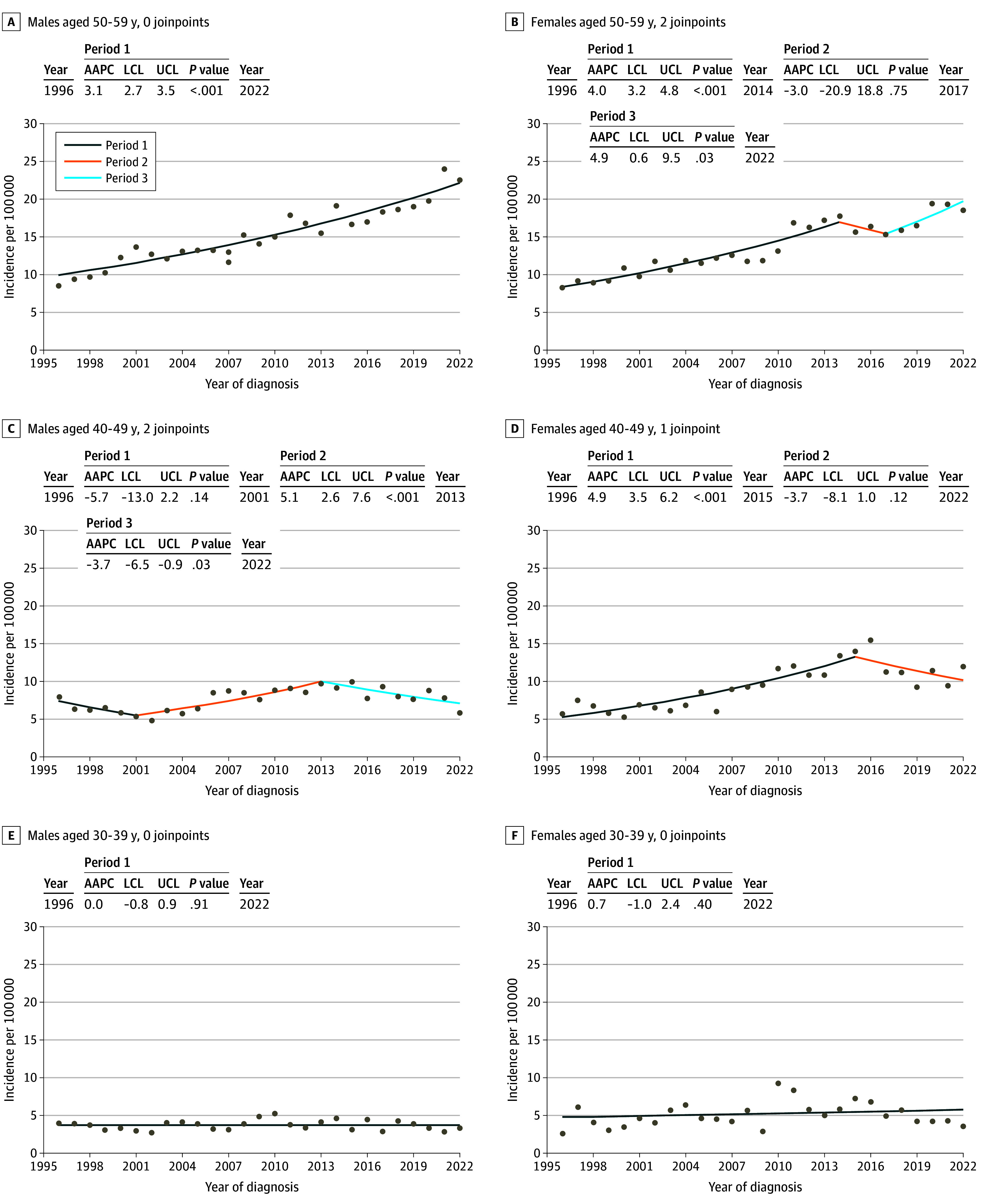
Average Annual Percentage Change (AAPC) in Incidence of Melanomas With Breslow Thickness Greater Than 1 mm in Sweden From 1996 to 2022, by Age Group Based on melanomas registered to the Swedish Melanoma Registry in 1996 to 2022. The year indicates the year of start and end of each period; LCL, lower confidence limit; and UCL, upper confidence limit.

Breslow tumor thickness data were available for 32 508 cases (93.4% of the total). Figure 2 shows the trend for melanomas 1 mm or thinner or thicker than 1 mm. Figure 3 shows the joinpoint analyses for melanomas thicker than 1 mm for ages 30 to 59 years, while eFigure 4 in Supplement 1 shows these results for ages 0 to 29 years. In the 50- to 59-year-olds, there was a significant increase in the incidence of thicker melanomas, with an AAPC of 4.9% (95% CI, 0.6 to 9.5;* P *= .029) and 3.1% (95% CI, 2.7 to 3.5;* P *< .001) in females and males, respectively, from 2017 to 2022. In the 40- to 49-year-olds, there was a significant increase in melanomas thicker than 1 mm until 2013 in males, and until 2015 in females. After that, a significant decrease was observed in females (AAPC, −3.7; 95% CI, −8.1 to 1.0;* P *= .12) and males (AAPC, −3.7%; 95% CI, −6.5 to −0.9;* P *= .01) alike. In the age groups up to 39 years, there were no significant changes or joinpoints for melanomas thicker than 1 mm.

### Melanoma Mortality Trends

**Figure 4.**
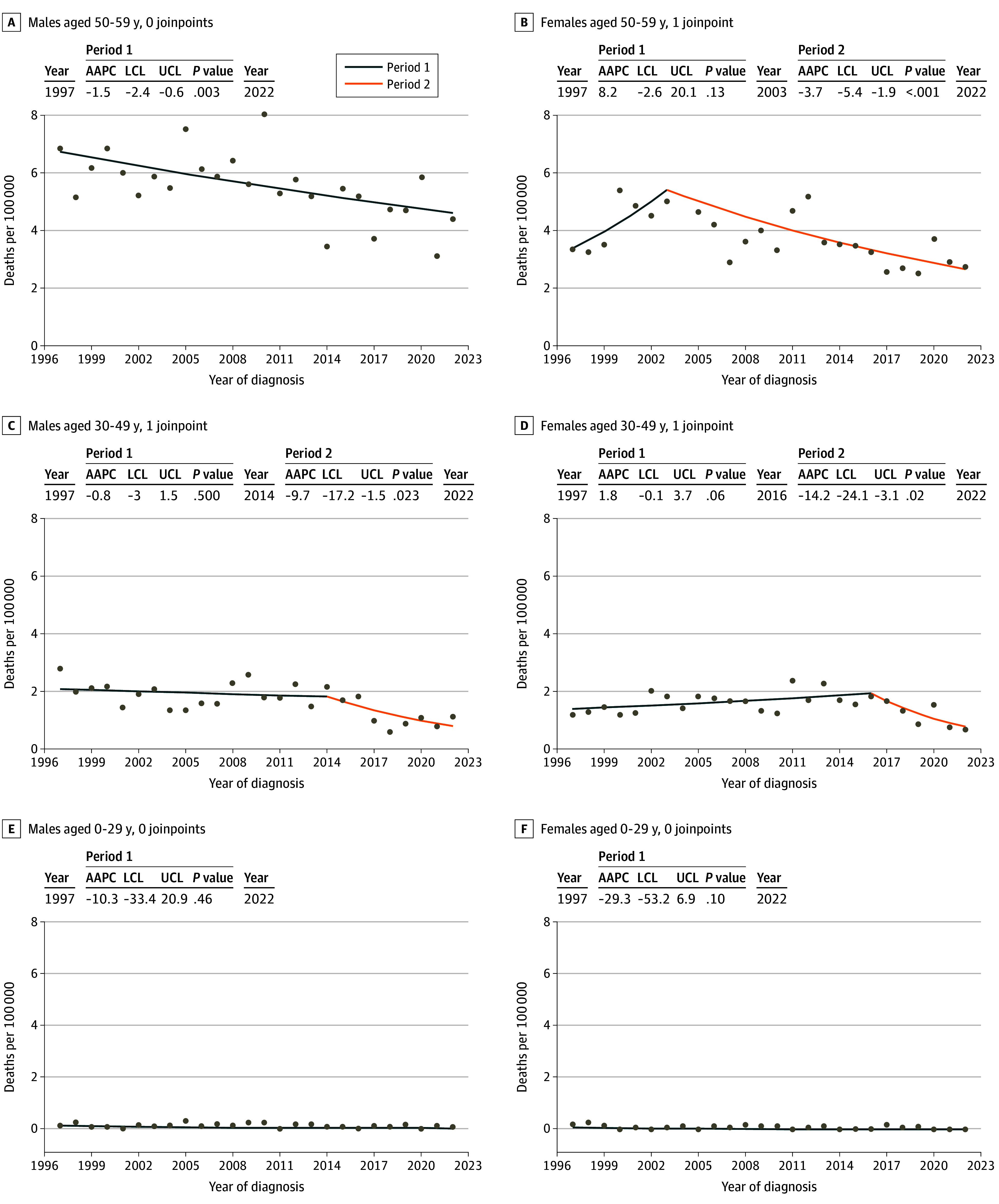
Mortality Trends and Average Annual Percentage Change (AAPC) in Melanoma Deaths in Sweden From 1997 to 2022, by Age Group Based on melanoma deaths registered to the Cause of Death Registry in Sweden from 1997 to 2022. The year indicates the year of start and end of each period; LCL, lower confidence limit; and UCL, upper confidence limit.

Trends in melanoma mortality and joinpoint analyses are shown in Figure 4. Mortality trends for the Swedish population 60 years and older are shown in eFigure 5 in Supplement 1. While there was a notable increase in the melanoma mortality in this older group, particularly in males, this was not the situation among the younger age groups. In the 50- to 59-year-olds, there was a significant decrease in melanoma mortality—in the males, the AAPC was −1.5 (95% CI, −2.4 to −0.6;* P *= .003), with no significant joinpoints, but the decline was most obvious from 2010 onward. In the 50- to 59-year-old females, there was a peak in 2003 followed by a significant decline in melanoma mortality (AAPC, −3.7; 95% CI, −5.4 to −1.9;* P *< .001). At the end of the study period, annual melanoma mortality was 5 per 100 000 in males and 3 per 100 000 in females for the age group from 50 to 59 years. Among those aged 30 to 49 years, mortality rates were stable until 2014 in males and until 2016 in females, and then declined signigicantly (AAPC in females, −14.2; 95% CI, −24.1 to −3.1;* P *= .02, and in males, −9.7; 95% CI, −17.2 to −1.5;* P *= .02 . At the end of the study period, annual melanoma mortality in the age group 30 to 49 years was 1 per 100 000. For the combined age group from 0 to 29 years, there were no significant joinpoints or changes over time, with an annual melanoma less than 0.1 per 100 000.

## Discussion

In this study from Sweden, focusing on melanoma trends among individuals younger than the average age of onset, we see that the incidence of invasive cutaneous melanomas has remained low in children and adolescents. From adolescence to 49 years, melanoma incidence was consistently higher among the females, which has also been reported as a consistent finding in other countries.^23^ In the female cohorts of 20- to 29- and 30- to 39-year-olds, we observed melanoma incidence surged, peaking in 2014 and then declining significantly. Similarly, incidence peaks were seen in males aged 30 to 39 years in 2014, and in both females and males aged 40 to 49 years in 2015. These peaks were followed by a nonsignificant downward trend. Interestingly, among 40- to 49-year-olds, there was a decline in melanomas thicker than 1 mm beginning in 2013 in males and in 2015 in females. Conversely, the age group from 50 to 59 years (slightly younger than the national median age of melanoma onset) followed the recognized population trend,^14^ with a persistent rise in melanoma incidence involving all melanoma thicknesses (≤1 mm and >1 mm).

For an epidemiologic perspective, the pervading trend break in melanoma incidence in Sweden among young adults from 2013 to 2015 is noteworthy. Given that this study is presenting only incidence over time from an epidemiologic perspective, we can only speculate on the underlying causes. The authors of this article are involved on the national level in developing guidelines and routines regarding accessibility to care, criteria for excision, histological diagnosis, and registration.

To our knowledge, there have been no substantial changes in these practices before or after the 2013 to 2015 period that would explain the trend change in young adults. A factor that has been suggested in this context is an association with immigration.^12,24,25^ Race is a variable that is not included in Swedish registries, but most Swedish residents are White and of Scandinavian descent, with Fitzpatrick skin types I to II being the most frequent phototype. However, since 2000, there has been a growing immigrant population, from 12% to currently, 27%, of the Swedish population is composed of first- or second-generation immigrants (defined as having been born outside of Sweden or having 2 parents born in another country) (eTable 1 in Supplement 1).^26^ Yet, half of the immigrant population are from other European countries, and hence only approximately 15% of residents of Sweden have origins from beyond Europe. Of the melanomas diagnosed in Sweden in 1990 to 2007, only 1% occurred in individuals from outside of Europe.^27^ Compared with the native Swedish population, the immigrant population pyramid comprises considerably younger individuals (eFigure 5 in Supplement 1).^26^ Among 20- to 49-year-olds, 35% currently have immigrant origins, compared with 22% among 60- to 69-year-olds (eTable 1 in Supplement 1). Hence, the higher frequency of immigrants among the younger adults could have contributed importantly to changes in melanoma incidence trends among the age groups from 20 to 49 years.

Comparison of the incidence peak in Sweden in 2013 to 2015 to a peak in melanoma incidence in Australia in the late 1990s among young adults could yield interesting insights.^9^ In response to its high skin cancer rates, Australia has led the way in public campaigns to promote sun protection and awareness of the dangers of UV radiation (UVR). In 1980, the country launched its *Slip! Slop! Slap!* campaign (slip on a shirt, slop on sunscreen, slap on a hat), which achieved high nationwide awareness.^28^ In Sweden, some activities promoting primary and secondary prevention started in the late 1980s, but it was in 1995 that the Swedish Cancer Society and the National Radiation Safety Authority launched the first comparable national campaign *Sola sakta* (caution in the sun) that particularly emphasized awareness among parents about protecting children from UVR using sunscreen, clothing, shade, and other methods to reduce exposure. One interpretation of how these campaigns affected trends is that protective behavior first becomes evident on a population level after approximately 20 years, and that the primary gain of UV protection so far has been in children and youth. Another factor that may have contributed is that legislation and recommendations by the National Radiation Safety Authority of Sweden have led to diminished access to indoor tanning devices over the past 2 to 3 decades.^29^

Our findings showed that in addition to declining incidence among those aged 30 to 49 years, there was also a significant decrease in melanoma mortality, which likewise started in 2014 to 2016. However, among those aged 50 to 59 years, there was a more constant and significant decline in mortality throughout the study period; notably, this was in contrast to the ongoing rise in melanoma incidence for this age group. There are several potential explanations for this mortality decrease, one of which is that increased incidence in the 50- to 59-year olds was predominantly presenting with thinner melanomas (≤1 mm thickness) that rarely metastasize. Moreover, Breslow thickness of 1 or less mm or greater than 1 mm is a rather blunt distinction given that, for example, 4-mm melanomas are most prone to metastasize but incidence of these has been stable. Additionally, death caused by melanoma usually occurs some years after diagnosis of the primary melanoma, and hence, the decrease in mortality among 50- to 59-year-olds may partly reflect the decrease in incidence in the younger age groups in the recent years. Additionally, since 2011, effective oncologic treatments have been available that significantly increase melanoma-specific survival—eg, immune checkpoint inhibitors and *BRAF* and *MEK* inhibitors.^30,31,32,33,34^ In the mortality analysis, we included those aged 60 years and older, and found no sign of a mortality decrease (Figure 3). A recent report using the World Health Organization global cancer database^15^ demonstrated a down trending in melanoma mortality in many countries, including Australia, the US, and the United Kingdom, particularly since 2010. Furthermore, a recent study from Sweden,^35^ also based on SweMR, found that in all age groups and in all primary melanoma stages melanoma-specific survival had increased from 1990 to 2020, with a more prominent increase since 2011. The lack of mortality improvement in those 60 years and older may be associated with the ongoing steep increase in incidence of melanomas, including those thicker than 4 mm, in the older population in Sweden.^14^

### Strengths and Limitations

An important strength of this study was the use of highly comprehensive registry data that allowed near-complete coverage of the Swedish population. The study also had limitations, including that melanoma in young individuals is relatively rare, and hence, the power for the trend analyses, particularly among children and adolescents, is quite low. Another limitation was that we present only epidemiologic data on incidence and mortality, and therefore, any causality regarding the risk factors mentioned are hypothetical. Another limitation was that we did not have access to race data; therefore, we cannot hypothesize whether the decreased incidence may be associated with demographic traits that may put the younger population in Sweden at a lower risk of melanoma.

## Conclusions

To summarize, in this nationwide cohort study of the Swedish population, we found a significant trend shift, with an incidence decline among younger individuals. These findings raise the hope that individuals 50 years and older will eventually follow suit, with lower melanoma incidence and mortality in the whole population. Raised public awareness, particularly about protecting children from sun exposure has possibly had as positive association with melanoma incidence and mortality in young adults. However, further studies are needed to address causal factors, including UVR exposure patterns and more detailed analysis of any associations with immigration and ancestry in the Swedish population.

## Notes

Supplement 1. eFigure 1. Incidence trends for primary invasive cutaneous melanoma in Sweden in males and females under the age of 60 years, per 100,000 in the years 1990-2022 subdivided by age groups eFigure 2. Average annual percentage change (AAPC) in the primary invasive cutaneous melanoma incidence in age groups 0-19 and 20-29 years in the Swedish population, from 1990 to 2022 eFigure 3. Incidence trends in Sweden in the years 1996-2022 for cutaneous melanomas with a Breslow thickness ≤1 mm or >1 mm eFigure 4. Average annual percentage change (AAPC) in the incidence of melanomas over 1 mm in thickness, in age groups 0-19 and 20-29 years in the Swedish population, from 1996 to 2022 eFigure 5. Mortality trends in males and females per 100,000 in the years 1997-2022 subdivided by age groups. eFigure 6. Population pyramid in the Swedish population in the year 2023 eTable. Swedish inhabitants in 2023 with foreign or Swedish background

Supplement 2. Data Sharing Statement
