# Supplementary material for: Melanoma Incidence and Mortality Trends Among Patients Aged 59 Years or Younger in Sweden
Source: JAMA Dermatol. 2024 Sep 8;160(11):1201–10. doi: 10.1001/jamadermatol.2024.3514 (PMC11382132; doi:10.1001/jamadermatol.2024.3514)
Supplement: Supplement 1. — eFigure 1. Incidence trends for primary invasive cutaneous melanoma in Sweden in males and females under the age of 60 years, per 100,000 in the years 1990-2022 subdivided by age groups eFigure 2. Average annual percentage change (AAPC) in the primary invasive cutaneous melanoma incidence in age groups 0-19 and 20-29 years in the Swedish population, from 1990 to 2022 eFigure 3. Incidence trends in Sweden in the years 1996-2022 for cutaneous melanomas with a Breslow thickness ≤1 mm or >1 mm eFigure 4. Average annual percentage change (AAPC) in the incidence of melanomas over 1 mm in thickness, in age groups 0-19 and 20-29 years in the Swedish population, from 1996 to 2022 eFigure 5. Mortality trends in males and females per 100,000 in the years 1997-2022 subdivided by age groups. eFigure 6. Population pyramid in the Swedish population in the year 2023 eTable. Swedish inhabitants in 2023 with foreign or Swedish background [file jamadermatol-e243514-s001.pdf]

Supplemental Online Content

Helgadottir H, Mikiver R, Schultz K, et al. Melanoma Incidence and Mortality Trends in Sweden. *JAMA Dermatol.* Published online September 8, 2024.
doi:10.1001/jamadermatol.2024.3514

eFigure 1. Incidence trends for primary invasive cutaneous melanoma in Sweden in males and females under the age of 60 years, per 100,000 in the years 1990-2022 subdivided by age groups

eFigure 2. Average annual percentage change (AAPC) in the primary invasive cutaneous melanoma incidence in age groups 0-19 and 20-29 years in the Swedish population, from 1990 to 2022

eFigure 3. Incidence trends in Sweden in the years 1996-2022 for cutaneous melanomas with a Breslow thickness ≤1 mm or >1 mm

eFigure 4. Average annual percentage change (AAPC) in the incidence of melanomas over 1 mm in thickness, in age groups 0-19 and 20-29 years in the Swedish population, from 1996 to 2022

eFigure 5. Mortality trends in males and females per 100,000 in the years 1997-2022 subdivided by age groups.

eFigure 6. Population pyramid in the Swedish population in the year 2023

eTable. Swedish inhabitants in 2023 with foreign or Swedish background

This supplemental material has been provided by the authors to give readers additional information about their work.

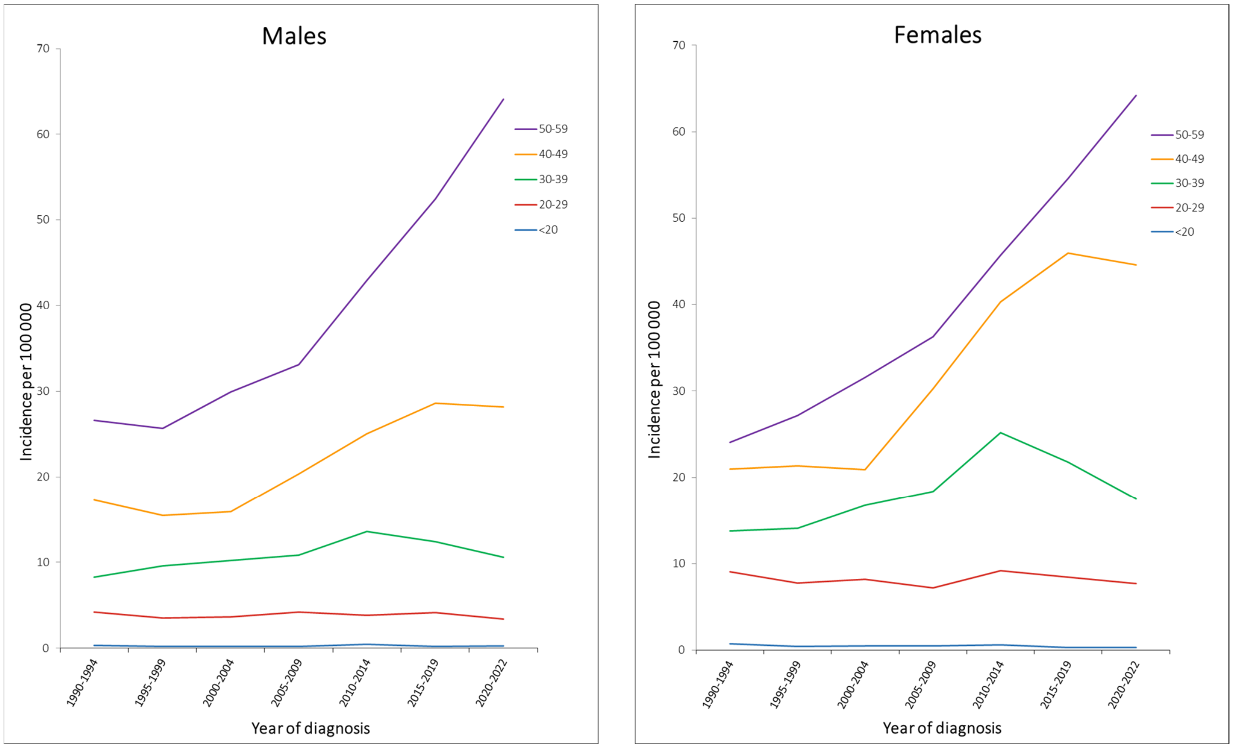

eFigure 1. Incidence trends for primary invasive cutaneous melanoma in Sweden in males and females under the age of 60 years, per 100,000 in the years 1990-2022 subdivided by age groups. Incidence is shown as the average incidence in every 5-year period (3-year period for the years 1990-2022). The figure is based on melanomas registered to the Swedish Cancer Registry in 1990-2022.

©2024 Helgadottir H et al. *JAMA Dermatol.*

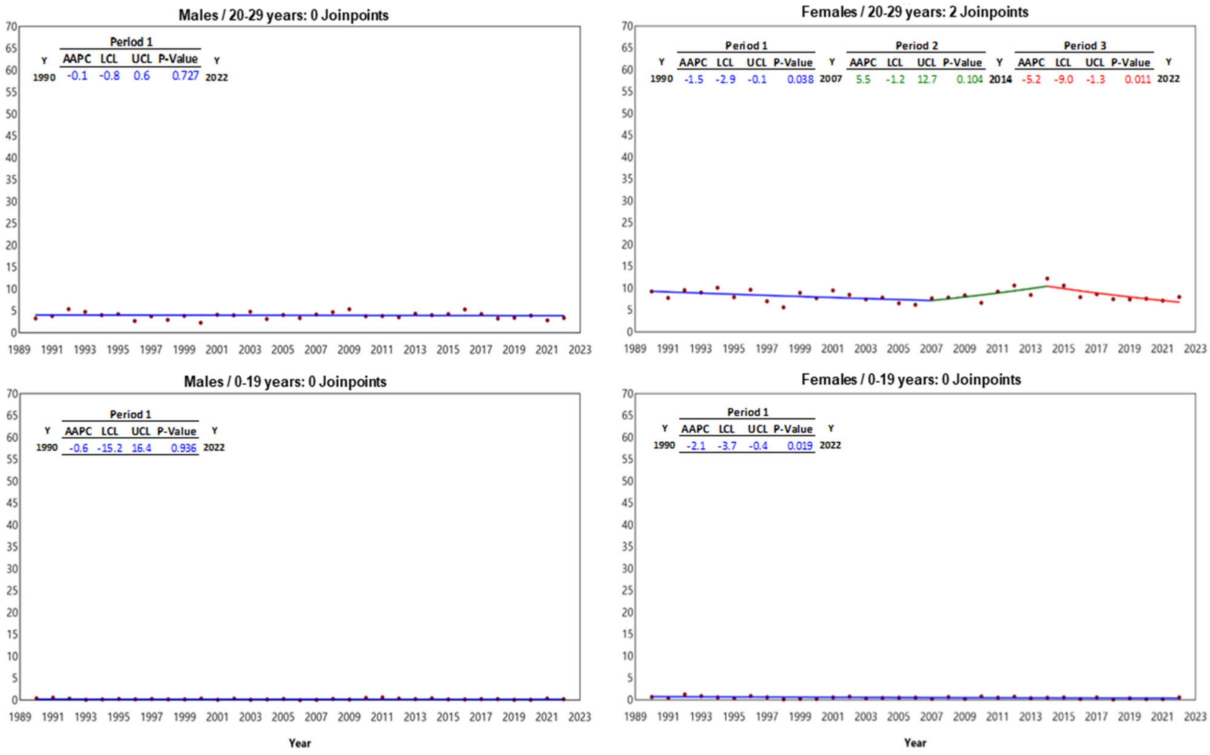

eFigure 2. Average annual percentage change (AAPC) in the primary invasive cutaneous melanoma incidence in age groups 0-19 and 20-29 years in the Swedish population, from 1990 to 2022. Y: year of start and end of each period. LCL: lower confidence limit, UCL: upper confidence limit. Y-Axis: Incidence per 100.000. X-Axis: Year of diagnosis. The figure is based on melanomas registered to the Swedish Cancer Registry in 1990-2022.

©2024 Helgadottir H et al. *JAMA Dermatol.*

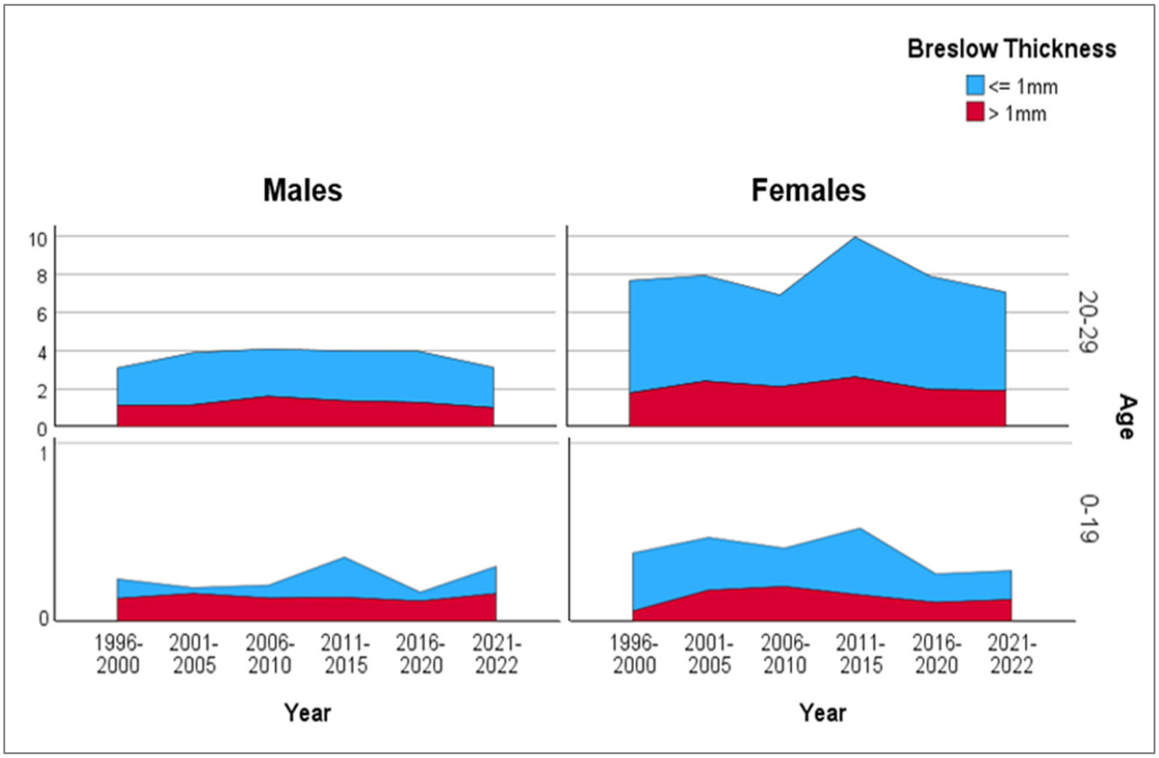

eFigure 3. Incidence trends in Sweden in the years 1996-2022 for cutaneous melanomas with a Breslow thickness ≤1 mm or >1 mm. Incidence is shown as the average incidence in every 5-year period (2-year period for the years 2021-2022). The figure is based on melanomas registered to the Swedish Melanoma Registry in 1996-2022. Scale adopted for the lower incidence in melanomas diagnosed up to 19 years (scale maximum 1 per 100,000) and 20-29 years (scale maximum 10 per 100,000).

©2024 Helgadottir H et al. *JAMA Dermatol.*

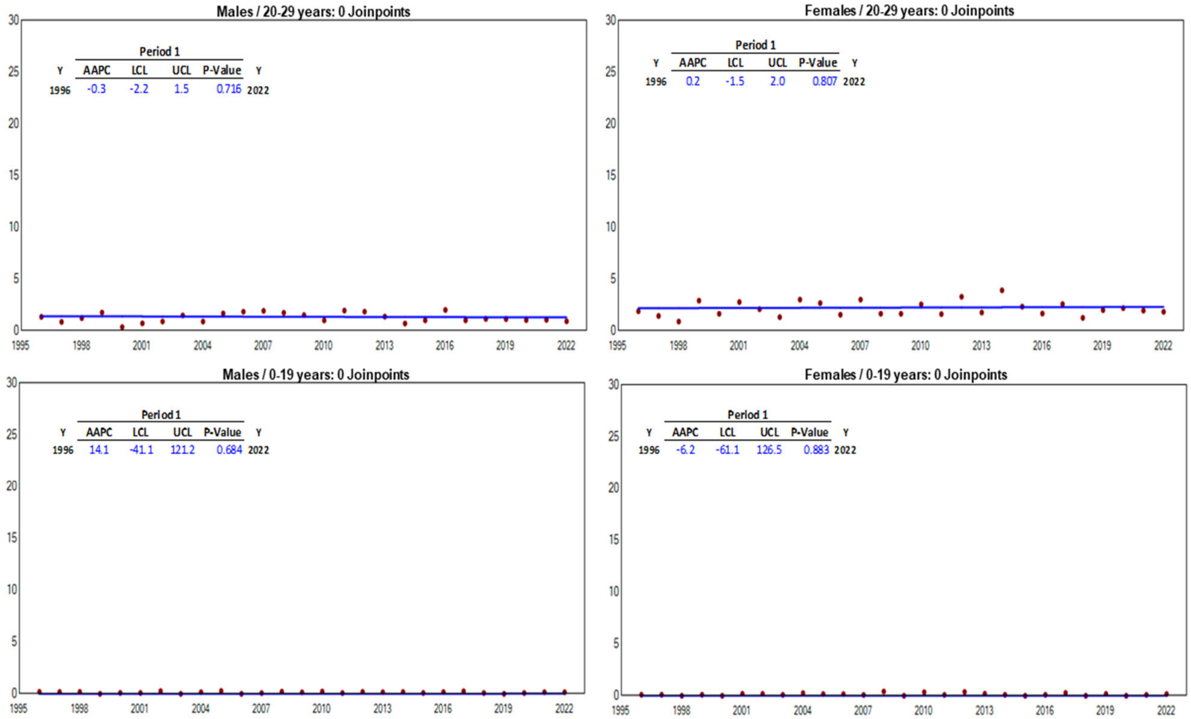

eFigure 4. Average annual percentage change (AAPC) in the incidence of melanomas over 1 mm in thickness, in age groups 0-19 and 20-29 years in the Swedish population, from 1996 to 2022. Y: year of start and end of each period. LCL: lower confidence limit, UCL: upper confidence limit. Y-Axis: Incidence per 100.000. X-Axis: Year of diagnosis. The figure is based on melanomas registered to the Swedish Melanoma Registry in 1996-2022.

©2024 Helgadottir H et al. *JAMA Dermatol.*

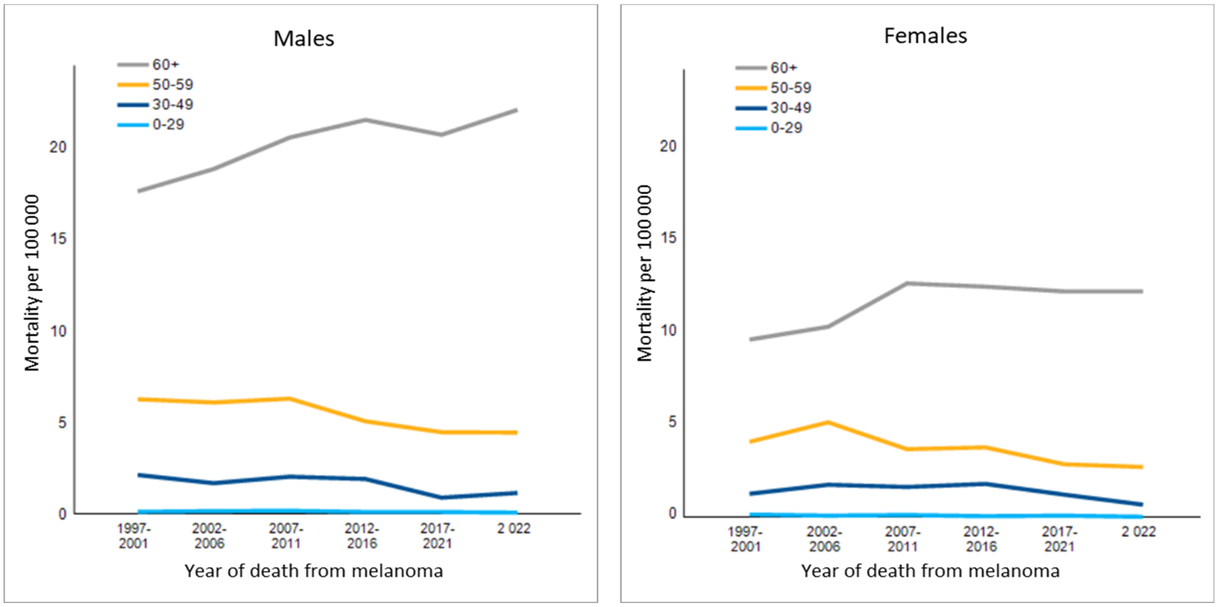

eFigure 5. Mortality trends in males and females per 100,000 in the years 1997-2022 subdivided by age groups. The figure is based on deaths from melanoma registered to the Cause of Death Registry in 1997-2022.

©2024 Helgadottir H et al. *JAMA Dermatol.*

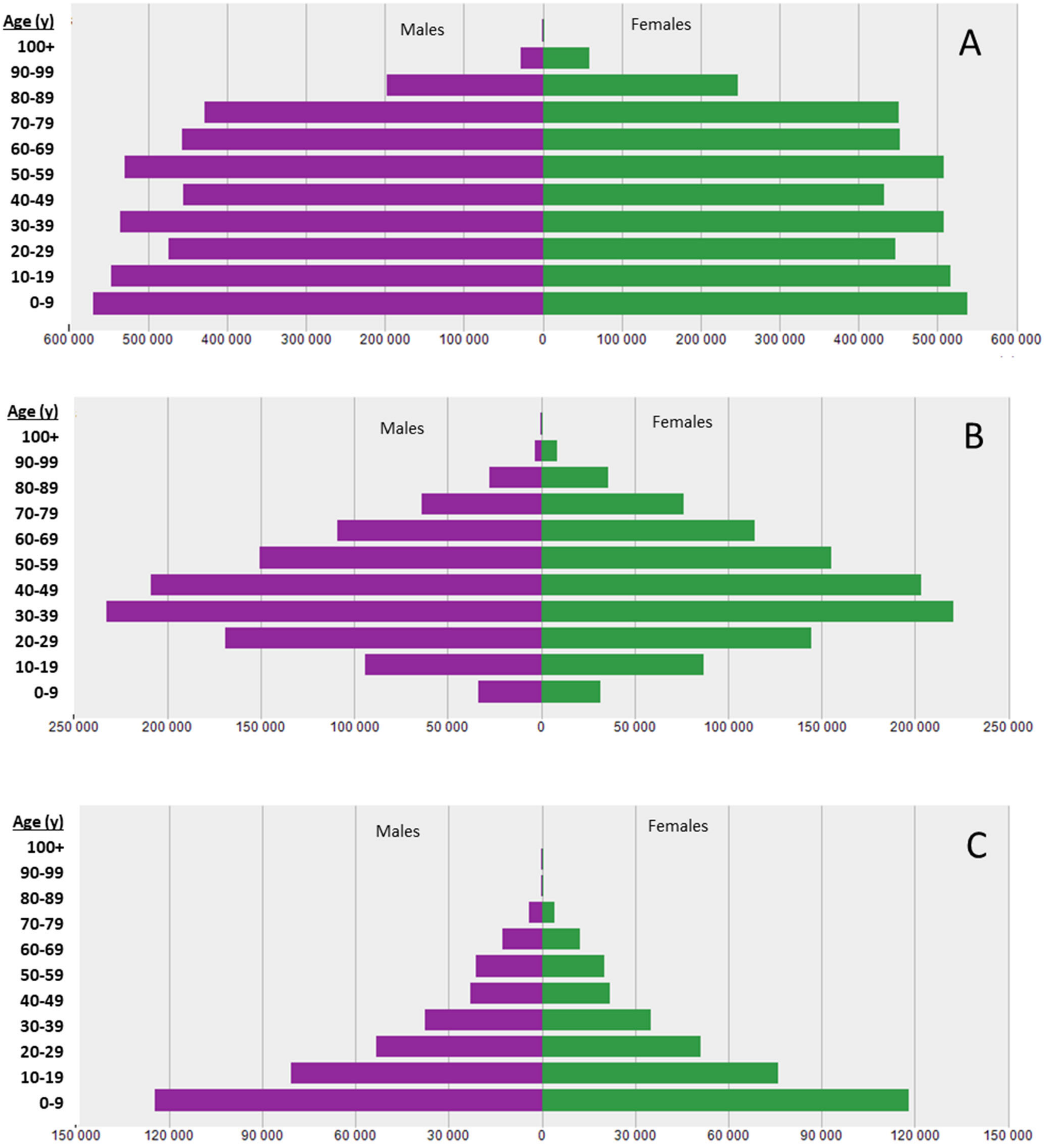

eFigure 6. Population pyramid in the Swedish population in the year 2023. A. Inhabitants born in Sweden. B. Inhabitants born outside Sweden (first-generation immigrants). C. Inhabitants where both parents are born outside Sweden (second-generation inhabitants). Source: Statistics Sweden, Statistiska Centralbyrån (SCB).

©2024 Helgadottir H et al. *JAMA Dermatol.*

eTable. Swedish inhabitants in 2023 with foreign or Swedish background

	Total population (n)	Foreign background* (n)	Swedish background** (n)	Fraction w. foreign background (%)
0-9 years				
Males	602,970	158,266	444,704	26.2%
Females	569,965	149,561	420,404	26.2%
10-19 years				
Males	640,276	174,946	465,330	27.3%
Females	603,048	162,970	440,078	27.0%
20-29 years				
Males	643,027	222,403	420,624	34.6%
Females	591,483	195,480	396,003	33.0%
30-39 years				
Males	767,918	269,914	498,004	35.1%
Females	728,210	255,691	472,519	35.1%
40-49 years				
Males	663,671	231,993	431,678	35.0%
Females	636,084	225067	411017	35.4%
50-59 years				
Males	679,899	171,814	508,085	25.3%
Females	663,206	175,746	487,460	26.5%
60-69 years				
Males	565,166	121,628	443,538	21.5%
Females	565,751	126,608	439,143	22.4%
70-79 years				
Males	492,371	67,976	424,395	13.8%
Females	527,106	80,354	446,752	15.2%
80-89 years				
Males	224,928	27,689	197,239	12.3%
Females	283,944	36,433	247,511	12.8%
90-99 years				
Males	31,817	3,326	28,491	10.5%
Females	68,117	8,478	59,639	12.4%
100+ years				
Males	476	59	417	12.4%
Females	2,274	274	2,000	12.0%
TOTAL	10,551,707	2,866,676	7,685,031	27.2%

*Defined as persons who are foreign born (first generation immigrant) or born in Sweden with both parents foreign born (second generation immigrant).

**Defined as persons who are born in Sweden with one or both parents born in Sweden

Source: Statistics Sweden, Statistiska Centralbyrån (SCB).

©2024 Helgadottir H et al. *JAMA Dermatol.*

Filename: DOI240040supp1_edited.docx
Directory: C:\Users\sthollan\Documents
Template:
C:\Users\sthollan\AppData\Roaming\Microsoft\Templates\Normal.dot
m
Title:
Subject:
Author: Hildur Helgadottir
Keywords:
Comments:
Creation Date: 8/27/2024 12:07:00 PM
Change Number: 2
Last Saved On: 8/27/2024 12:11:00 PM
Last Saved By: SR Holland
Total Editing Time: 0 Minutes
Last Printed On: 8/27/2024 12:12:00 PM
As of Last Complete Printing
Number of Pages: 8
Number of Words: 770 (approx.)
Number of Characters: 4,390 (approx.)
