# Supplementary material for: Melanoma Incidence and Mortality Trends Among Patients Aged 59 Years or Younger in Sweden
Source: JAMA Dermatol. 2024 Sep 8;160(11):1201–10. doi: 10.1001/jamadermatol.2024.3514 (PMC11382132; doi:10.1001/jamadermatol.2024.3514)
Supplement: Supplement 2. — Data Sharing Statement [file jamadermatol-e243514-s002.pdf]

Data Sharing Statement

Helgadottir. Melanoma Incidence and Mortality Trends in Sweden. *JAMA Dermatol*. Published September 09, 2024. doi:10.1001/jamadermatol.2024.3514

Data
Data available: No

Additional Information
Explanation for why data not available: We are not allowed to share information on patients or outcomes from Swedish Health and Population Registries.
